# Dual Assessment of Developmental Topographical Disorientation: Comparing Self-Reported Measures with Actual Navigational Performance

**DOI:** 10.3390/brainsci15030318

**Published:** 2025-03-18

**Authors:** Alessia Bonavita, Sofia Pepe, Raffaella Nori, Massimiliano Palmiero, Cecilia Guariglia, Laura Piccardi

**Affiliations:** 1 Faculty of Law, Giustino Fortunato University, 82100 Benevento, Italy; 2 Psychology Department, Sapienza University of Rome, 00185 Rome, Italy; sofia.pepe@uniroma1.it (S.P.); cecilia.guariglia@uniroma1.it (C.G.); 3 Psychology Department, University of Bologna, 40100 Bologna, Italy; raffaella.nori@unibo.it; 4 Department of Communication Sciences, University of Teramo, 64100 Teramo, Italy; mpalmiero@unite.it; 5 Cognitive and Motor Rehabilitation and Neuroimaging Unit, IRCCS Fondazione Santa Lucia, 00179 Rome, Italy; 6 Department of Medicine and Health Sciences, University of Molise, 86100 Campobasso, Italy; 7 Cassino San Raffaele Hospital, 03043 Cassino, Italy

**Keywords:** developmental topographical disorientation, anxiety, SOD, spatial orientation, topographic orientation, map-based navigation, landmark knowledge, individual differences, psychological distress

## Abstract

**Background/Objectives:** This study aimed to (i) evaluate the effectiveness of the Familiarity and Spatial Cognitive Style Scale (FSCS) and the short Computerized Ecological Navigational Battery (LBS) in predicting navigational performance by comparing self-reported scores with actual results; (ii) investigate the FSCS’s potential as a screening tool for Developmental Topographical Disorientation (DTD), which affects about 3% of youth, focusing on early detection; and (iii) examine gender differences in self-reported data versus real-world performance to understand how stereotypes affect self-assessment. **Methods**: The study involved 185 college students (125 female), aged 18–35 years, who completed the FSCS and performed navigation tasks using a new version of the LBS. Participants’ performances were analysed using MAD-based z-scores to identify potential DTD cases, with scores below the fifth percentile flagged for further investigation. The relationship between self-reported abilities and actual performance was assessed through correlation analyses and robust linear regressions. **Results**: The SOD subscale of FSCS emerged as a comprehensive predictor of navigation performance, correlating significantly with accuracy across multiple tasks. The study identified a 5.42% prevalence of DTD using FSCS criteria, aligning with previous research, while LBS identified 11.96% of participants with navigational difficulties. Gender differences were observed in Survey Knowledge and Landmark Ordering tasks, with males showing higher performances. Only two participants were flagged as DTD cases by both assessment methods, suggesting they may evaluate distinct aspects of navigational ability. **Conclusions**: The findings validate FSCS as an initial screening tool for DTD while highlighting the need for comprehensive assessment using multiple tools. The study suggests the existence of at least two distinct forms of DTD: one affecting navigational memory (detectable by both FSCS and LBS) and another impacting perceptual navigation aspects (more readily identified by LBS). These results emphasise the importance of developing a detailed DTD taxonomy and implementing personalised interventions based on specific navigational challenges.

## 1. Introduction

Effectively navigating one’s surroundings often means seeking guidance from others or consulting a map. For example, as a tourist eager to explore the city, one might ask hotel staff for recommendations or refer to a map for accurate directions. This choice will probably depend on an individual’s ability to navigate. Some people are naturally better at navigation and instinctively know their exact location, no matter the circumstances. Individuals can be categorized as skilled (good) or unskilled (poor) navigators, with a spectrum of abilities between these two extremes. In addition, some individuals are diagnosed with Developmental Topographic Disorientation (DTD), a condition first identified in 2009 by Iaria et al. [1] and subsequently reported more consistently in other studies [2,3,4], manifesting as a specific learning disorder related to environmental navigation.

Undoubtedly, navigation depends on internal and external factors that affect the way in which individuals explore the environment itself [5,6]. Internal factors include cognitive styles, which refer to individuals’ tendencies to notice specific environmental cues over others (field dependence/independence) [7,8], sex [9,10,11] (with men considered more skilled than women), age [12,13,14,15,16,17,18,19] (navigational skills tend to develop in adolescence and may decline with age), familiarity with the environment [20,21,22], and job-related expertise [23,24,25,26,27]. External factors refer to environmental configuration and contextual elements such as landmarks, circulation systems, and signage [28,29,30].

Both internal and external factors contribute to what people call their sense of direction (SOD), which can be conceptualised as a cognitive awareness of the ability to recognise and comprehend precise location or orientation within a given environment [31]. According to Sholl, Acacio, Makar, and Leon [32], SOD is an understanding of one’s body position in relation to important landmarks and geographical features on Earth. Given that self-perception plays a crucial role in both definitions, it is understandable that the majority of SOD assessment tools rely on self-assessment. These instruments invite individuals to evaluate their own ability to navigate and orientate themselves within their environment. Kozlowski and Bryant’s [31] SOD scale asks, “How good is your sense of direction?” Participants respond using a Likert-type rating scale, where 1 signifies “poor” and 7 signifies “good”. In an advancement, Hegarty, Montello, Richardson, Ishikawa, and Lovelace [33] introduced the 15-item Santa Barbara Sense of Direction Scale, designed to assess this important construct comprehensively. In 2011, Piccardi, Risetti, and Nori [34] developed the Familiarity and Spatial Cognitive Style Scale (FSCS), a comprehensive 22-item assessment tool. This scale incorporates various internal factors and offers a cut-off point for identifying individuals affected by DTD. DTD is a neurodevelopmental disorder that impairs individuals’ ability to navigate their environment despite the absence of neurological or psychiatric conditions. Those with DTD have been affected since birth, and the challenges typically become evident during adolescence as they strive for greater independence in their daily movements [2,3].

Individuals with DTD typically exhibit normal memory and neuropsychological profiles; however, they experience significant difficulties in spatial cognition. They often report profound challenges in everyday navigation, struggling to utilise cognitive maps or location-based strategies to navigate both familiar and new surroundings effectively.

They often face significant challenges with metric spatial encoding [4]. Many individuals struggle to advance beyond basic landmark knowledge, finding it difficult to associate verbal labels with spatial directions linked to specific landmarks or to create a mental map of a location, even if it is familiar to them.

DTD is increasingly common, impacting approximately 3% of the young population [4]. Therefore, access to a reliable tool for assessing an individual’s sense of direction is essential in early detection and intervention. Without timely treatment, affected individuals may experience psychological distress that can escalate to social withdrawal.

Claessen and van der Ham [35] have proposed a new model to classify topographical deficits. They propose that navigational impairments may concern landmark knowledge, location knowledge, and path knowledge domains. Spatial knowledge is acquired by recognising distinct, noticeable landmarks linked through personal sensory and directional information. This helps in forming routes and creating a cognitive map that includes both directional and distance information about the environment. According to these authors, impairment in landmark-based navigation involves an inability to encode, retrieve, or recognise salient landmarks or environmental scenes, both novel and familiar, while impairments in location-based navigation entail an inability to acquire or recall environmental knowledge about landmark locations and their spatial relations both from an egocentric and allocentric perspective in both familiar and novel environments [35]. Impairments in path-based navigation are complex, involving difficulty in forming or retrieving information about how locations are connected. This affects the use of route or survey knowledge in both familiar and new environments. Furthermore, landmark-based and path-based navigation impairments can occur together or separately, while location-based navigation impairments occur only with the other two types [35,36].

Studies have demonstrated that people’s self-evaluations of their navigational skills serve as highly reliable predictors of their wayfinding abilities [1,2,3,4,37,38]. Prestopnik and Roskos-Ewoldsen [38] established that SOD predicts wayfinding ability. However, Nori and Piccardi [39] uncovered that SOD may be influenced by negative stereotypes regarding women’s navigational skills. Their study revealed that women tend to self-assess their SOD more poorly than men. Yet, when navigating familiar environments, women demonstrate equal proficiency in wayfinding to their male counterparts.

This finding may clarify why Iaria and Barton [2] reported a higher prevalence of DTD among women than men, while other studies found an equal proportion of men and women suffering from DTD [4,40]. It may result in varying impacts, influenced by negative stereotypes across diverse cultures. Negative stereotypes have a substantial effect on making decisions about careers and general interests [41]. Although women have a 25% lower likelihood of being involved in a car accident [42], general opinion considers women less skilled in driving behaviour, and the impact of this stereotype produces negative psychological effects during the driving experience, especially anxious reactions [43].

This study had multiple objectives:

1. Assess the FSCS and the short version of the Computerised Ecological Navigational Battery (LBS) as reliable predictors of actual navigational performance by contrasting self-reported scores with outcomes from a navigational battery.
2. Investigate the FSCS’s effectiveness as a screening tool for DTD, a condition with an increasing prevalence that affects approximately 3% of youth, highlighting the significance of early detection.
3. Analyse potential gender differences in self-reported SOD versus real-world navigational performance to address the impact of negative stereotypes on self-assessment.

This investigation holds particular importance as the FSCS can represent a first-step test in identifying individuals at risk for DTD. Early and accurate detection can help mitigate the psychological distress and social withdrawal often linked with untreated navigational difficulties.

## 2. Materials and Methods

### 2.1. Participants

The original sample included 295 participants; however, several exclusions were necessary. Fifty individuals were excluded for not completing the FSCS and LBS, not finishing the anamnestic questionnaire, or stating that they were familiar with Latina city. Additionally, 35 participants were disqualified due to the presence of psychiatric disorders such as psychosis, depression, schizophrenia, or panic attacks. Furthermore, 20 participants had sustained brain injuries, including head trauma or perinatal injuries, while 5 individuals reported issues related to substance abuse.

The final sample included 185 college students (female = 125) recruited through volunteer enrolment from the Sapienza University of Rome. Participants ranged in age from 18 to 35 years (mean = 21.00, SD = 3.30) and had an average educational level of 13.73 years (SD = 1.53). The sample was equally balanced by gender (χ^2^(183) = 184.00, *p* = 0.465). All participants have normal or corrected-to-normal (soft contact lenses or glasses) vision. We assessed both right- and left-handers to ensure a representative examination of DTD within the general population. Each participant met the inclusion criteria specified in the anamnestic questionnaire. Specifically, we excluded participants with neurological and psychiatric disorders, as well as those with addiction behaviour (for additional details, see the Instruments subsection). All participants came from and grew up in urban areas, knew the topographic codes of orientation, and were fully familiar with these codes. Written informed consent was obtained from all participants before their involvement in the study. The research adhered to the ethical principles outlined in the Declaration of Helsinki, and ethical approval was obtained from the Ethical Committee of the I.R.C.C.S. Fondazione Santa Lucia in Rome.

### 2.2. Instruments

#### 2.2.1. The Anamnesis Questionnaire

The Anamnesis Questionnaire [4] gathers information through a comprehensive series of questions addressing various issues related to spatial orientation difficulties encountered since childhood. The inquiry delved into potential neurological or significant psychiatric history, prior traumatic brain injuries, learning disabilities, and substance abuse, including alcohol and drugs. Concerning neurological outcomes, the questionnaire evaluates incidents of head trauma, ischaemic attacks, encephalitis, brain infections, and complications during the prenatal and perinatal stages. On the psychiatric front, it explores conditions such as depression, anxiety, psychosis, obsessive–compulsive disorder, eating disorders, post-traumatic stress disorder, schizophrenia, and phobias. Additionally, it collects data on the duration of these conditions, any ongoing pharmacological treatments, and the individual’s current medication status. Additionally, it assesses the frequency of alcohol consumption and inquires whether the respondent has ever used or currently uses drugs, including substances like cannabis, amphetamines, and cocaine. If the respondent answers affirmatively, then the questionnaire delves into details regarding the specific substances utilised, including the timeline and frequency of their use.

The Anamnesis Questionnaire is administered to exclude participants who report neurologic or psychiatric disorders, as well as individuals who abuse substances.

#### 2.2.2. Familiarity and Spatial Cognitive Style Scale (FSCS)

Assessment using the Familiarity and Spatial Cognitive Style Scale (FSCS) [34,44] is made up of several subscales that include Sense of Direction (SOD), Town Knowledge (TK), Spatial Cognitive Style (SCS, including Landmark, Route, and Survey), and Right–Left Confusion (RLC). Each item is rated on a Likert scale, with 1 indicating low-level ability and 5 indicating high-level ability. An individual is classified as having DTD if their total SOD score falls 2 standard deviations (SDs) below the mean reported by Nori and Piccardi) [44]. To diagnose DTD, we considered the SOD subscale and the four diagnostic criteria proposed by Iaria and Barton (2010) [2]: (i) frequently becoming lost (1 to 5 times a week) in familiar environments; (ii) experiencing spatial orientation problems since an early age; (iii) the absence of any other cognitive difficulties that may impact daily activities; and (iv) no known brain lesions, malformations, or conditions affecting the central nervous system, except migraines. In addition, we considered two additional criteria: (v) no active psychiatric disorders or use of psychotropic drugs and (vi) no substance abuse behaviour. These added criteria are investigated through the Anamnesis Questionnaire.

#### 2.2.3. The Short Version of the Computerised Ecological Navigational Battery (LBS)

**Figure 1:**
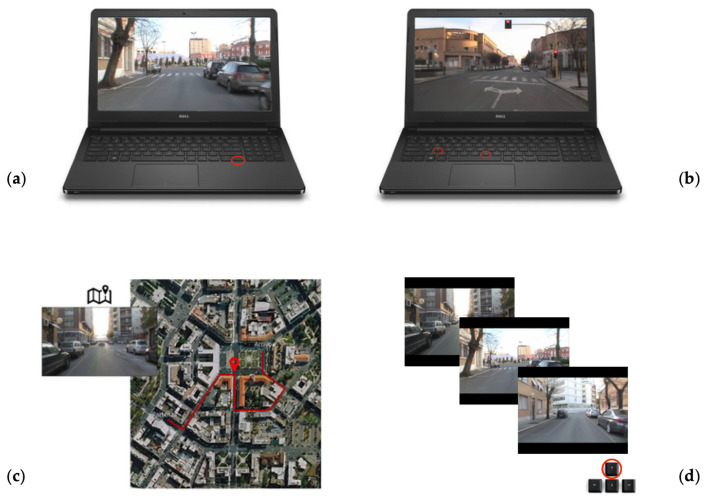
(**a**) The image shows an example of the Route Knowledge task. Participants must choose the correct direction to take upon reaching each crossroads. (**b**) The image shows an example of the Landmark Knowledge task. Participants have to indicate whether the displayed image is part of the previously seen path by pressing ‘S’ for yes and ‘N’ for no on the keyboard. (**c**) The image shows an example of stimuli for the Survey Knowledge task. Participants have to choose whether the position of the placeholder on the map corresponds to the position of the crossroads shown in the upper left corner. (**d**) The image shows an example of the Landmark Ordering task. Participants have to indicate whether the crossroads shown precedes or follows the previously displayed crossroads by using the directional arrows on the keyboard: up for precedes and down for follows.

The short version of the Computerised Ecological Navigational Battery (LBS) [45,46] reflects the intricate nature of spatial navigation in an ecological setting (see Figure 1). It facilitates the assessment of one’s capacity to acquire and utilise various forms of spatial knowledge regarding a new environment by recording both accuracy (the total number of correct responses) and response times. The LBS battery consists of several subtests, beginning with Route Knowledge (RK). In this initial subtest, participants view a video capturing a journey from a first-person perspective, as if they were behind the wheel. The video pauses at every intersection along the route, prompting participants to indicate the car’s left, right, or straight direction by pressing the corresponding directional arrows on their keyboard. The video is shown three times and provides feedback about the accuracy of responses. In the initial attempt, responses are provided at random. However, in the second and third attempts, participants must recall and respond based on the correct answers that they learned earlier. Accuracy in the third attempt is measured with a maximum score of 9. In the Landmark Knowledge (LK) subtest, participants observe an image of a crossroad displayed prominently at the centre of the screen. They must determine whether the picture depicts one of the crossroads that they encountered along the route by pressing the S key for “yes” or the N key for “no”. The maximum accuracy for this subtest is 18. During the Survey Knowledge (SK) subtest, participants view a satellite map of the test environment at the centre of the screen, with the learned path highlighted by a red line. In the upper left corner, an image of one of the crossroads is shown, along with a placeholder along the path correlating with that crossroads. Participants must indicate whether the placeholder accurately represents the position of the displayed crossroad by pressing the S key for “yes” or the N key for “no”. The maximum accuracy for this subtest is also 18. The last Landmark Ordering (LO) subtest is a 1-back task where images of crossroads are presented in a continuous sequence at the centre of the screen. Participants must determine whether the currently displayed crossroad comes before or after the previous one by pressing the forward arrow (for “after”) or the backward arrow (for “before”) on the keyboard. If the crossroad is identical to the previous one, they should press the spacebar. The maximum accuracy for this subtest is 72. For this study, we created a new version of the LBS battery that was identical in structure to the original but with a different set of stimuli (Figure 1).

### 2.3. Data Analysis

All statistical analyses were conducted using Jamovi (Version 2.6; The Jamovi Project, 2022, https://www.jamovi.org assessed on 20 August 2024), a user-friendly open-source statistical platform. Descriptive statistics were calculated for all variables to evaluate central tendencies and variability. The Shapiro–Wilk test was performed to assess the normality of the data distributions, with results indicating significant deviations from normality (Table 1).

To assess the reliability of the new version of LBS, we executed the internal consistency check for each task. Afterwards, we performed Spearman’s Rho correlation analysis to examine the relationships between FSCS and LBS performance. We then performed a series of robust linear regressions to assess whether FSCS scores, specifically the SOD subscale and the total score, predict navigational performance in the various subtests of the LBS battery. Each regression used accuracy in one of the LBS subtests as the outcome variable, with FSCS scores as predictors.

The Median Absolute Deviation (MAD) method, as described by [47], was applied to compute MAD-based z-scores for all variables. This transformation, known for its robustness, facilitated the comparison of participants’ performance on tasks with differing units of measurement. MAD-based z-scores represent a robust technique for non-normal data distributions to minimise the influence of extreme values [47,48,49]. To prevent division by zero (or tiny numbers) in the z-score calculation, a small constant (epsilon = 0.01) is added to the denominator. This constant is sufficiently small to avoid distorting the data while ensuring that the variance remains non-zero.

We then flagged individuals with potential DTD based on their performance in the LBS, focusing on their accuracy and response time. Higher accuracy scores and lower response times reflect better performance in these tasks. We first computed the 5th and 95th percentiles of the MAD-based Z-scores for each variable to identify atypical performance and establish a reference range for typical performance. Individuals whose MAD-based Z-scores fell outside this range—either below the 5th percentile or above the 95th percentile—were flagged as demonstrating atypical performance. We summed the flags across all tasks for each participant to aggregate these findings. If the sum of flags exceeded 1, then the participant was classified as a potential case of DTD, suggesting that their performance on multiple tasks was sufficiently atypical to warrant further consideration. We then proceeded to apply the same methodology to the SOD subscale of the FSCS. Scores below the 5th percentile of MAD-based Z-scores were flagged as DTD, indicating atypically low SOD scores (Table 2). This approach followed standard outlier detection practices, where extreme values far from the central tendency are flagged for further investigation [47,50]. Subsequently, given the demographic similarities between our participants, we aimed to assess whether our DTD criteria were consistent with those reported by Piccardi et al. [4], who identified a 3% prevalence of DTD among a large group of young Italian adults. For SOD-based flagging, 9 participants were identified as DTD cases, resulting in a prevalence of 5.42%. A z-test for proportions was conducted to compare this rate to the 3% prevalence reported by Piccardi et al. [4]. While the observed prevalence in our study was higher, the difference was not statistically significant (z = 1.829, *p* = 0.067), indicating that the higher prevalence in our sample could be due to random variation and aligned with the previous study’s findings. In contrast, 22 participants were flagged for the LBS-based flagging for DTD, resulting in a prevalence rate of 11.96%, significantly higher than the 3% reported by Piccardi et al. [4]. A statistical analysis using a z-test for proportions revealed a substantial difference, with a z-value of 7.122 (*p* < 0.001), indicating that the higher prevalence in our study is unlikely to be due to random chance.

To evaluate the presence of gender differences across the FSCS subscales and LBS performance metrics, we initially conducted a Kruskal–Wallis ANOVA and a pairwise comparison using the Dwass–Steel–Critchlow–Fligner test to explore better significant differences.

## 3. Results

### 3.1. LBS New Version Reliability

Internal consistency was assessed for each task to evaluate the reliability of the newly adapted version of the LBS. The RK task showed acceptable internal consistency (α = 0.671), while the LK task demonstrated moderate reliability (α = 0.623). The SK task exhibited good internal consistency (α = 0.731), and the LO task revealed very high internal consistency (α = 0.956). We then performed a robust linear regression analysis to examine the relationships between the tasks. We used the LO task as the dependent variable and performance in the RK, LK, and SK tasks as independent predictors. SK significantly predicted performance in the LO task (β = 0.362, *t* = 6.456, *p* < 0.001), as well as performance in the RK task (β = 0.001, t = 3.56, *p* < 0.001). However, LK did not significantly predict performance in the LO task (β = 0.00045, t = 1.29, *p* = 0.198). Although, in this new version, we found that the RK task played a role in the prediction of LO task performances, these findings align overall with the original version of the LBS and confirm the findings of previous studies [45,51].

### 3.2. Relationships Between FSCS Scores and Navigational Performance

**Figure 2:**
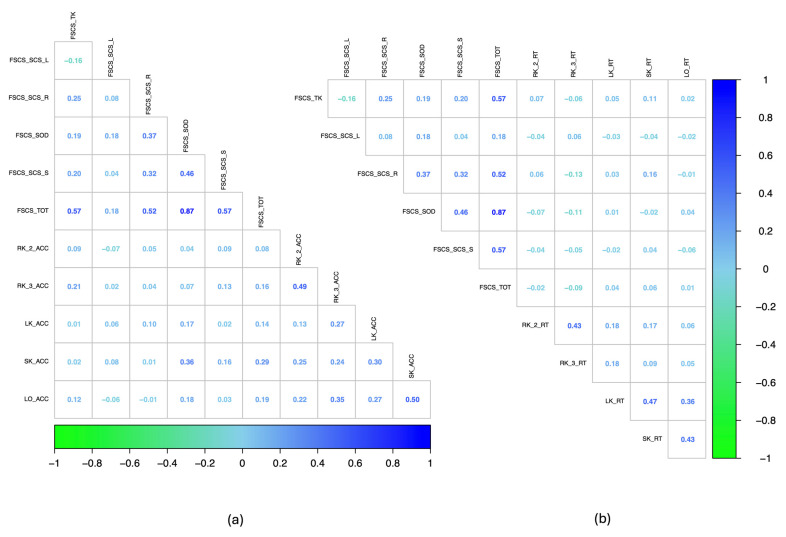
(**a**) The left panel shows the correlogram representing the correlation matrix between the FSCS scores and the LBS accuracy scores across all subtests. (**b**) The right panel shows the correlogram representing the correlation matrix between the FSCS scores and the LBS response time across all subtests; FSCS_SCS_L, the score obtained in the landmark spatial cognitive style subscale of the Familiarity and Spatial Cognitive Style Scale; the FSCS_SCS_R, the score obtained in the Route Spatial Cognitive Style subscale of the Familiarity and Spatial Cognitive Style Scale; FSCS_SCS_S, the score obtained in the Survey Spatial Cognitive Style subscale of the Familiarity and Spatial Cognitive Style Scale; FSCS_SOD, the score obtained in the Sense of Direction subscale of the Familiarity and Spatial Cognitive Style Scale; FSCS_TOT, the total score of the Familiarity and Spatial Cognitive Style Scale; RK_2, route knowledge at the second attempt; RK_3, route knowledge at the third attempt; LK, landmark knowledge; SK, survey knowledge; LO, landmark ordering knowledge; ACC, accuracy; RT, response time.

We first performed Spearman’s Rho correlation analysis to examine the relationships between FSCS and LBS performance. The results show that FSCS is positively associated with key navigational abilities. The TK subscale correlates significantly with RK accuracy at the third attempt (ρ = 0.215, *p* = 0.005). The SOD subscale showed positive correlations with the LK accuracy (ρ = 0.171, *p* = 0.028), SK accuracy (ρ = 0.355, *p* < 0.001), and LO accuracy (ρ = 0.118, *p* = 0.023). The Route SCS scale was associated with the SK reaction time (ρ = 0.160, *p* = 0.040). The total FSCS score demonstrated significant associations with the RK accuracy at the third attempt (ρ = 0.157, *p* = 0.044), the SK accuracy (ρ = 0.287, *p* < 0.001), and the LO accuracy (ρ = 0.188, *p* = 0.016). There were no significant correlations with the LBS response times. These findings suggest that FSCS, particularly the SOD subscale, may serve as a reliable predictor of actual navigation performance, emphasising its role in accuracy across multiple navigational measures (Figure 2).

**Figure 3:**
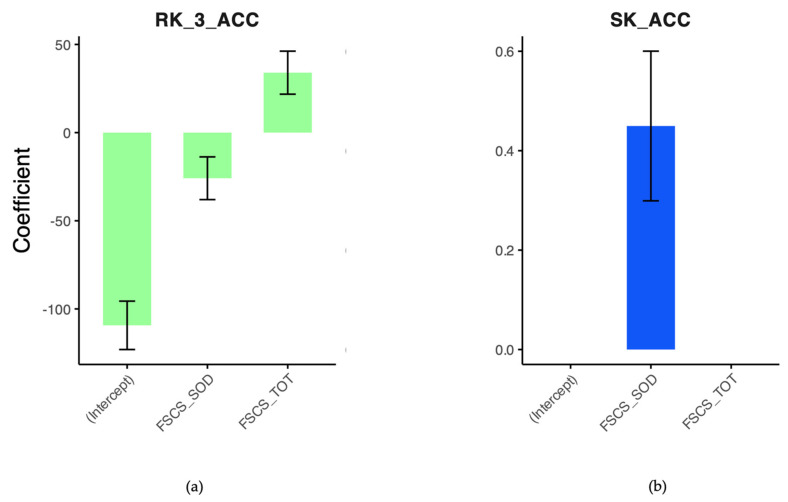
(**a**) The graph shows the significant predictors for route knowledge at the third attempt. (**b**) The graph shows the significant predictors for survey knowledge. RK_3_ACC, accuracy in route knowledge at the third attempt; SK_ACC, accuracy in survey knowledge; FSCS_SOD, the score obtained on the Sense of Direction subscale of the Familiarity and Spatial Cognitive Style Scale; FSCS_TOT, the total score on the Familiarity and Spatial Cognitive Style Scale.

We then performed a series of robust linear regressions to assess whether FSCS scores, specifically the SOD subscale and the total score, predict navigational performance in the various subtests of the LBS battery. Each regression used accuracy in one of the LBS subtests as the outcome variable, with FSCS scores as predictors. The results revealed no significant relationships for the RK subtest at the second attempt (Intercept: β = −0.432, t = −2.251, *p* = 0.024; FSCS_SOD: β = −0.159, t = −0.769, *p* = 0.442; FSCS_TOT: β = 0.216, t = 1.133, *p* = 0.257), the LK subtest (Intercept: β = −198.211, t = −9.849, *p* < 0.001; FSCS_SOD: β = 16.620, t = 0.850, *p* = 0.396; FSCS_TOT: β = 0.395, t = 0.021, *p* = 0.984), or the LO subtest (Intercept: β = −0.180, t = −1.077, *p* = 0.281; FSCS_SOD: β = 0.115, t = 0.865, *p* = 0.387; FSCS_TOT: β = 0.046, t = 0.383, *p* = 0.702). However, significant predictors emerged for the RK subtest at the third attempt, where FSCS_SOD (β = −25.88, t = −2.136, *p* = 0.033) and FSCS_TOT (β = 33.99, t = 2.787, *p* = 0.005) were associated with performance. Additionally, in the SK subtest, FSCS_SOD significantly predicted performance (β = 0.450, t = 2.988, *p* = 0.003), while FSCS_TOT did not (β = −0.102, t = −0.700, *p* = 0.484). These findings suggest that FSCS scores, particularly the SOD subscale, may play a significant role in predicting performance in tasks requiring spatial knowledge (Figure 3).

### 3.3. Predictive Utility of FSCS Scores for Identifying DTD

We evaluated the DTD flagging methods of the SOD subscale and the LBS to examine their agreement and assess the FSCS’s effectiveness as a screening tool for DTD. Initially, we conducted a cross-tabulation analysis to compare participants flagged as DTD by the SOD subscale (*n* = 9) and the LBS tasks (*n* = 19). Among the 166 participants analysed, 140 were not flagged as DTD by either method, while 17 were flagged exclusively by LBS, and 7 were flagged exclusively by SOD. Only two participants were flagged as DTD by both methods. The z-test for differences in proportions showed no significant association between the methods (χ^2^(1) = 1.044, *p* = 0.296), supported by Fisher’s exact test (*p* = 0.275). Effect size measures, including the Phi coefficient (φ = 0.081) and Cramer’s V (V = 0.081), indicated weak agreement between the two methods. Cohen’s Kappa revealed poor agreement between SOD and LBS in identifying DTD cases. The unweighted and weighted Kappa coefficients were 0.075 (95% CI: −0.11 to 0.26), suggesting negligible agreement. These results imply that SOD and LBS may assess different dimensions of navigational difficulties, with SOD capturing self-perceived navigational ability and LBS evaluating objective performance. We then conducted McNemar’s test to evaluate whether the discrepancy between cases flagged by SOD and LBS was significant. The continuity-corrected chi-squared value (χ^2^(1) = 3.375, *p* = 0.066) revealed a marginally non-significant trend, suggesting that SOD identified fewer DTD cases than LBS. This finding supports the hypothesis that SOD may operate with stricter thresholds or assess a construct distinct from that measured by LBS.

### 3.4. Impact of Gender

We initially conducted a Kruskal–Wallis ANOVA to evaluate gender differences across the FSCS subscales and LBS performance metrics. The results revealed significant gender differences in SK accuracy (χ^2^(1) = 6.533, ρ = 0.011, ε^2^ = 0.036, males: M = 16.2, females: M = 14.8) and LO accuracy (χ^2^(1) = 8.539, ρ = 0.003, ε^2^ = 0.047, males: M = 45.6, females: M = 40.9) for the LBS tasks, indicating that males and females might demonstrate distinct spatial performance patterns. A significant difference was also observed for the SOD subscale (χ^2^(1) = 3.796, ρ = 0.050, ε^2^ = 0.023, males: M = 38.2, females: M = 36.8), whereas no significant differences were found for the other FSCS subscales. Pairwise comparisons using the Dwass–Steel–Critchlow–Fligner test revealed significant differences in SK accuracy, with males performing better than females (W = 3.615, *p* = 0.011). For LO accuracy, males again outperformed females significantly (W = 4.133, *p* = 0.003). No significant pairwise differences were observed for the SOD subscale (W = 2.755, *p* = 0.051).

To evaluate gender differences in DTD flagging, we performed a Kruskal–Wallis ANOVA. The analysis for DTD status based on LBS tasks revealed no significant gender differences (χ^2^(1) = 0.995, *p* = 0.319, ε^2^ = 0.005). Similarly, no significant gender differences were found for DTD status based on the SOD subscale (χ^2^(1) = 0.508, *p* = 0.476, ε^2^ = 0.003). These results indicate that gender does not significantly influence the likelihood of being flagged as DTD by either method, suggesting that the flagging criteria are equally applicable across genders.

We then conducted a cross-tabulation analysis to compare DTD flagging across methods within each gender. Among females, SOD flagged 7.2% of participants as DTD compared to 14.4% flagged by LBS, while among males, SOD flagged 1.8% compared to 5.4% flagged by LBS, reflecting a smaller difference. These findings indicate potential gender-related variations in the sensitivity and specificity of the two methods. McNemar’s test was applied for each gender. For females, McNemar’s test showed a non-significant difference (χ^2^(1) = 2.5, *p* = 0.1), while Cohen’s Kappa (κ = 0.0054, 95% CI: −0.17 to 0.18) demonstrated negligible agreement between methods. Similarly, for males, McNemar’s test revealed no significant difference (χ^2^(1) = 0.25, *p* = 0.6), with a slightly higher Cohen’s Kappa value (κ = 0.3, 95% CI: −0.2 to 0.8), suggesting low agreement. These results indicate that while there are some gender-related differences in the percentage of cases flagged by each method, the overall agreement between SOD and LBS remains weak across both genders.

## 4. Discussion

The present study focused on assessing the reliability and clinical utility of the FSCS for evaluating an individual’s SOD ability, as well as the LBS. Specifically, our study had a threefold purpose: (i) assessing the FSCS as a reliable predictor of actual navigational performance by contrasting self-reported scores with outcomes from a navigational battery; (ii) investigating the FSCS’s effectiveness as a screening tool for DTD; and (iii) analysing potential gender differences in self-reported SOD versus real-world navigational performance to address the impact of negative stereotypes on self-assessment.

### 4.1. Evaluating the FSCS as a Predictor of Navigational Performance with Respect to LBS

Concerning LBS, results evidenced good reliability and internal consistency for key subtests of the battery, such as LO and SK, reinforcing the robustness of this assessment tool. Furthermore, SK significantly predicted performance on LO and RK tasks. However, our results also showed that RK plays a role in the prediction of LO performance, suggesting that RK may be more integrated with high-level navigational skills. This suggests a hierarchy in navigational skills, progressing from simple to complex, supporting the model of environmental knowledge acquisition proposed by Siegel and White in 1975 [52]. However, Survey-level representations might not always emerge after RK, but rather in parallel with it, depending on individual navigation experience and task demands [53,54]. This perspective is furthermore supported by evidence that the transition from RK to SK is managed by a distinct neural mechanism rather than a simple stepwise progression [26,55]. The predictive power of RK in our results may reflect the involvement of allocentric processing mechanisms, which, as suggested by neuroimaging studies, play a crucial role in both route-based and survey-based navigation [26,55,56,57].

In general, the new version of LBS is reliable, with consistent and strong reliability for the LO task. The predictive relationships largely align with previous research [45,51], though the significant role of RK is a new finding.

The SOD subscale of FSCS emerges as the most comprehensive predictor of navigation performance, correlating with accuracy across multiple tasks.

### 4.2. Investigating the FSCS’s Effectiveness as a Screening Tool for DTD

The results discussed above support the SOD subscale of FSCS as a screening tool for navigational abilities, especially considering its use to compute a first cut-off to identify potential cases with DTD [4,40]. In this vein, positive correlations emerge between self-reported SOD and navigational accuracy, suggesting that people generally rate their spatial orientation skills fairly well.

We compared individuals under the cut-off score in SOD of the FSCS and individuals with deficits in LBS. The data presented are intriguing, as FSCS and LBS assess distinct facets of navigation. FSCS operates as a self-assessment tool with a stringent cut-off designed to minimise false positives, which makes it less effective in exploring perceptual disturbances in navigation. In contrast, these disturbances may become more evident in a landmark navigation test like that used in LBS. This distinction aligns with previous research highlighting the involvement of different brain networks in allocentric versus egocentric navigation strategies. Specifically, landmark-based navigation relies on the parahippocampal place area (PPA) and retrosplenial cortex [58], while survey-based navigation requires broader hippocampal involvement [26]. These findings suggest that FSCS primarily captures subjective awareness of spatial abilities, whereas LBS objectively evaluates visuospatial and perceptual navigation processes. Only two participants were flagged by both methods, which strongly suggests that LBS and FSCS are two sides of the same coin, assessing, in a complementary manner, different aspects of navigational abilities.

These two participants consistently expressed difficulties with becoming lost, a challenge that they had faced from a young age. Data from FSCS largely confirmed data from Piccardi et al. [4] supporting the validity of this questionnaire in identifying individuals at risk for DTD. In contrast, the data from LBS showed that around 11% of the sample suffered from navigational disorders.

In summary, the SOD-based identification method successfully identified nine participants with DTD, resulting in a prevalence rate of 5.42%. This rate is comparable to the 3% prevalence reported by Piccardi et al. [4]. The lack of statistical significance between these two percentages indicates that the SOD-based identification method aligns with previous research outcomes, confirming that the FSCS is an excellent initial tool for guiding subsequent in-depth neuropsychological diagnostics. Moreover, the evidence indicates that LBS, an ecological computerised tool for measuring spatial navigation, effectively identifies more individuals with navigational disorders. This finding underscores the notion that spatial cognition is a multidimensional construct, requiring both self-reporting and objective performance-based methods to ensure a comprehensive assessment of all its components. These results align with proposals by Aguirre and D’Esposito [59], who suggested that topographical disorientation may consist of multiple subtypes, each affecting distinct components of spatial navigation. It further emphasises the urgent need to establish a taxonomy for DTD. Currently, it is evident that two distinct forms of DTD exist: one significantly affects navigational memory, as shown by both the FSCS and LBS tools, while the other impacts perceptual aspects of navigation, particularly landmark recognition, exemplified by the case of FG described by Piccardi et al. [60]. This second form is detected by LBS but is less evident with FSCS. However, it is possible that there is an additional subtype of DTD. To develop targeted intervention strategies aimed toward specific deficits, it is crucial to identify these subtypes, such as impairments in landmark recognition, route learning, or survey knowledge integration [61], or those linked to executive functions, such as travel planning and problem-solving [62]. Consequently, future research should aim to uncover additional types of navigational disorders, to refine DTD classification, taking into account a multimodal assessment to ensure a comprehensive understanding of individual differences in navigational impairments. This exploration will enable the creation of personalised training interventions tailored to the specific navigational challenges that individuals face.

### 4.3. Examining Gender Differences in SOD and Navigation Performance Regarding Negative Stereotypes

However, it is important to recognise that various psychological factors significantly influence how individuals evaluate their own abilities; indeed, self-perceived navigational competence can be influenced by cognitive biases, cultural norms, and individual differences in self-confidence [26,63,64,65]. A study conducted by Nori and Piccardi [39] highlighted a notable difference in self-assessment between men and women. Generally, men perceived themselves as more skilled and more knowledgeable about their surroundings than women did. However, when tasked with navigational challenges in a familiar environment, both groups performed equally well, demonstrating that women often experience a negative stereotype effect that impacts their self-perception. Research indicates that personal beliefs about differences in abilities between men and women can either hinder or enhance an individual’s performance [66,67].

Strong beliefs that men excel at specific tasks more than women (or vice versa) can lead to diminished performance among those perceived as less capable in the context of stereotyped activities [66,67]. The stereotype threat effect, in particular, has been shown to induce performance anxiety in individuals from groups expected to underperform, leading to self-doubt and impaired task execution [68,69,70,71,72]. Conversely, individuals from the group believed to have superior abilities experience a stereotype lift, enhancing their performance due to the influence of a positive stereotype [73]. Research in spatial cognition has revealed that gender stereotypes significantly impact performance in tasks related to mental rotation, with men often expecting to outperform women [69,74], and navigation [39,75,76,77]. However, these differences are often attributable to stereotype-related biases rather than inherent cognitive disparities, as women perform equally well when tested in stereotype-free conditions or in familiar environments [39,68].

In the present study, men were better than women in LBS, and they were more accurate than women in LO and SK. This result is consistent with previous research indicating that men tend to rely more on allocentric (survey-based) strategies, which involve mentally integrating spatial relationships from a global perspective, whereas women are more likely to adopt egocentric (landmark-based) strategies, which rely on recognising specific environmental cues [11,78]. We also found sex differences in FSCS, especially in the SOD subscale: men rated themselves as having a higher sense of direction than women did. This discrepancy aligns with previous findings suggesting that women tend to underestimate their navigational abilities compared to men, a bias that may be influenced by stereotype threat [9,74].

## 5. Limitations and Future Perspectives

This study, while providing valuable insights into spatial orientation and navigational abilities, has several limitations that should be acknowledged. First, the sample size, particularly when stratified by gender and compared across different assessment methods, may limit the generalizability of our findings. The relatively low agreement (κ = 0.3) between the SOD and LBS assessment methods suggests that our current approach to identifying navigational disorders may require refinement. Second, the study predominantly relied on computerised navigation tasks, which, despite efforts to maximise ecological validity, may not fully capture the complexity of real-world navigation. The artificial testing environment might not account for additional contextual factors that influence navigation in daily life, such as environmental complexity, time pressure, and emotional states. Third, while we examined the impact of gender stereotypes on self-reported navigation abilities, we did not implement specific experimental manipulations to isolate the stereotype threat effect. This limits our ability to establish causal relationships between stereotypes and performance differences. Fourth, our assessment focused primarily on specific aspects of navigation (landmark orientation, route knowledge, and survey knowledge), potentially overlooking other cognitive processes that contribute to navigational performance, such as working memory, executive functions, and visual attention. Fifth, the current study did not include neuroimaging techniques that could have provided valuable insights into the neural correlates of navigation abilities and their relationship with self-reported skills.

Future research should aim to address these limitations and extend our understanding of navigational abilities in several directions. First, developing a comprehensive taxonomy of DTD subtypes is critical. As our findings suggest, navigational disorders may manifest differently across individuals, affecting distinct components of spatial cognition. Larger-scale studies with diverse populations are needed to identify and characterise these subtypes with greater precision. Second, future studies should incorporate multimodal assessment approaches that combine self-report measures, objective performance-based tests, and neuroimaging techniques. This integrative approach would provide a more holistic understanding of the relationship between subjective awareness of spatial abilities and objective navigational performance, while also elucidating the underlying neural mechanisms. Third, developing more ecologically valid assessment tools that better simulate real-world navigation challenges would enhance the clinical utility of diagnostic measures. Virtual reality technologies offer promising opportunities to create immersive, realistic navigation environments while maintaining experimental control. Fourth, longitudinal studies tracking the development of navigational abilities across the lifespan would provide valuable insights into how these skills evolve and how intervention strategies could be optimised for different age groups. Fifth, future research should explicitly address the impact of stereotype threat on navigational performance through targeted experimental designs. This would help disentangle the contributions of gender-related stereotypes from actual cognitive differences and inform educational approaches to mitigate these effects. Sixth, developing personalised training interventions tailored to specific navigational deficits represents an important avenue for future work. Such interventions could target landmark recognition, route learning, survey knowledge integration, or executive function components of navigation, depending on individual needs. Finally, cross-cultural studies examining how navigational abilities and self-assessment vary across different cultural contexts would enrich our understanding of the social and environmental factors that shape spatial cognition. This knowledge would be valuable for developing culturally sensitive assessment tools and intervention strategies for diverse populations. By addressing these future directions, researchers can advance our understanding of spatial navigation abilities and develop more effective approaches to identifying and supporting individuals with navigational disorders.

## 6. Conclusions

The present study provides insights into the assessment of spatial orientation and navigation abilities through both self-report measures (the FSCS) and performance-based testing (the LBS).

The LBS showed strong reliability, particularly in the Landmark Ordering task, with Survey Knowledge emerging as a significant predictor of performance across other navigational components. This hierarchical relationship supports traditional models of environmental knowledge acquisition. Our findings demonstrate several key points about these complementary assessment tools and their implications for understanding DTD. The SOD subscale of the FSCS proved to be a robust predictor of actual navigation performance across multiple tasks, validating its utility as an initial screening tool for DTD.

Our findings highlight the existence of at least two distinct forms of DTD: one affecting navigational memory (detectable by both FSCS and LBS) and another impacting perceptual aspects of navigation, particularly landmark recognition (more readily identified by LBS). This distinction emphasises the need for a comprehensive DTD taxonomy and suggests that multiple assessment tools may be necessary for accurate diagnosis.

Our investigation revealed important gender-related findings, with men showing higher performance in LBS tasks (particularly in Landmark Ordering and Survey Knowledge) and reporting a higher self-rated sense of direction on the FSCS. However, these differences should be interpreted within the context of documented stereotype effects in spatial cognition, where negative stereotypes can impact women’s self-assessment and performance in navigational tasks.

## Figures and Tables

**Table 1:** Descriptive statistics for the LBS battery and the FSCS scores.

	Mean	Median	SD	Shapiro–Wilk	
				W	*p*
RK_2_ACC	7.59	8.00	1.60	0.82	<0.001
RK_2_RT	1585.08	1251.44	1310.18	0.53	<0.001
RK_3_ACC	8.29	9.00	1.26	0.62	<0.001
RK_3_RT	1577.94	1172.16	1552.78	0.50	<0.001
LK_ACC	17.02	18.00	1.48	0.68	<0.001
LK_RT	2536.17	2281.94	1527.34	0.57	<0.001
SK_ACC	14.53	15.00	2.95	0.92	<0.001
SK_RT	6708.08	6029.06	3588.44	0.85	<0.001
LO_ACC	46.89	49.50	16.65	0.96	<0.001
LO_RT	1973.36	1811.05	1622.84	0.59	<0.001
FSCS_TK	42.36	42.00	6.23	0.99	0.552
FSCS_SOD	37.60	37.00	9.47	0.99	0.194
FSCS_SCS_L	6.67	6.50	1.82	0.96	<0.001
FSCS_SCS_R	7.57	8.00	1.51	0.94	<0.001
FSCS_SCS_S	5.07	5.00	1.96	0.95	<0.001
FSCS_TOT	99.28	99.00	14.32	0.99	0.621

**Table 2:** Descriptive statistics for the potential DTD participants.

	DTD_Status	Sex	N	Mean	SD
Age	DTD_LBS	F	17	21.82	3.61
		M	5	21.60	3.44
	No DTD_LBS	F	108	20.41	2.83
		M	54	21.87	3.88
	DTD_SOD	F	7	20.143	2.035
		M	2	21.500	3.536
	No DTD_SOD	F	104	20.673	3.064
		M	53	21.736	3.768

## Data Availability

The original data presented in the study are openly available in OSF at the following DOI 10.17605/OSF.IO/THBY2.

## References

[1] Iaria G., Bogod N., Fox C.J., Barton J.J.S. (2009). Developmental topographical disorientation: Case one. Neuropsychologia.

[2] Iaria G., Barton J.J. (2010). Developmental topographical disorientation: A newly discovered cognitive disorder. Exp. Brain Res..

[3] Burles F., Iaria G. (2020). Behavioural and cognitive mechanisms of Developmental Topographical Disorientation. Sci. Rep..

[4] Piccardi L., Palmiero M., Cofini V., Verde P., Boccia M. (2022). ‘Where am I?’ A snapshot of the developmental topographical disorientation among young Italian adults. PLoS ONE.

[5] Gärling T., Böök A., Lindeberg E. (1986). Spatial orientation and way-finding in the designed environment. A conceptual analysis and some suggestions for postoccupancy evaluation. J. Archit. Plan. Res..

[6] Kitchin R.M. (1994). Cognitive maps: What are they and why study them?. J. Environ. Psychol..

[7] Li H., Zhang Y., Wu C., Mei D. (2016). Effects of field dependence-independence and frame of reference on navigation performance using multi-dimensional electronic maps. Personal. Individ. Differ..

[8] Boccia M., Vecchione F., Di Vita A., D’Amico S., Guariglia C., Piccardi L. (2019). Effect of Cognitive Style on Topographical Learning Across Life Span: Insights From Normal Development. Child Dev..

[9] Nazareth A., Huang X., Voyer D., Newcombe N. (2019). A meta-analysis of sex differences in human navigation skills. Psychon. Bull. Rev..

[10] Munion A.K., Stefanucci J.K., Rovira E., Squire P., Hendricks M. (2019). Gender differences in spatial navigation: Characterizing wayfinding behaviors. Psychon. Bull. Rev..

[11] Lawton C.A., Chrisler J., McCreary D. (2010). Gender, spatial abilities and wayfinding. Handbook of Gender Research in Psychology.

[12] Moffat S.D., Zonderman A.B., Resnick S.M. (2001). Age differences in spatial memory in a virtual environment navigation task. Neurobiol. Aging.

[13] Ramanoël S., York E., Le Petit M., Lagrené K., Habas C., Arleo A. (2019). Age-Related Differences in Functional and Structural Connectivity in the Spatial Navigation Brain Network. Front. Neural Circuits.

[14] Li A.W.Y., King J. (2019). Spatial memory and navigation in ageing: A systematic review of MRI and fMRI studies in healthy participants. Neurosci. Biobehav. Rev..

[15] Muffato V., Hilton C., Meneghetti C., Beni R.D., Wiener J.M. (2019). Evidence for age-related deficits in object-location binding during place recognition. Hippocampus.

[16] Fernandez-Baizan C., Diaz-Caceres E., Arias J.L., Mendez M. (2019). Egocentric and allocentric spatial memory in healthy aging: Performance on real-world tasks. Braz. J. Med. Biol. Res..

[17] Piccardi L., Nori R., Palermo L., Guariglia C. (2015). Age effect in generating mental images of buildings but not common objects. Neurosci. Lett..

[18] van der Ham I.J.M., Claessen M.H.G., Evers A.W.M. (2020). Large-scale assessment of human navigation ability across the lifespan. Sci. Rep..

[19] Reinoso-Medina L., Thrasher C.A., Harburger L.L. (2024). Evidence for age-related decline in spatial memory in a novel allocentric memory task. Neuropsychol. Dev. Cogn. B Aging Neuropsychol. Cogn..

[20] Iachini T., Ruotolo F., Ruggiero G. (2009). The effects of familiarity and gender on spatial representation. J. Environ. Psychol..

[21] Akbari K., Winter S., Tomko M. (2025). A causal analysis of environmental familiarity on navigation information needs. Int. J. Geogr. Inf. Sci..

[22] Nori R., Zucchelli M.M., Palmiero M., Piccardi L. (2023). Environmental cognitive load and spatial anxiety: What matters in navigation?. J. Environ. Psychol..

[23] Piccardi L., Palmiero M., Bocchi A., Boccia M., Guariglia C. (2019). How does environmental knowledge allow us to come back home?. Exp. Brain Res..

[24] Weisberg S.M., Newcombe N.S., Chatterjee A. (2019). Everyday taxi drivers: Do better navigators have larger hippocampi?. Cortex.

[25] Lopez A., Caffò A.O., Bosco A. (2018). Topographical disorientation in aging: Familiarity with the environment does matter. Neurol. Sci..

[26] Wolbers T., Hegarty M. (2010). What determines our navigational abilities?. Trends Cogn. Sci..

[27] Hegarty M., He C., Boone A.P., Yu S. (2023). Understanding differences in wayfinding strategies. Top. Cogn. Sci..

[28] Lawton C.A. (1996). Strategies for indoor wayfinding: The role of orientation. J. Environ. Psychol..

[29] Dogu U., Erkip F. (2000). Spatial factors affecting wayfinding and orientation: A case study in a shopping mall. Environ. Behav..

[30] Armougum A., Orriols E., Gaston-Bellegarde A., Joie-La Marle C., Piolino P. (2019). Virtual reality: A new method to investigate cognitive load during navigation. J. Environ. Psychol..

[31] Kozlowski L.T., Bryant K.J. (1977). Sense of direction, spatial orientation, and cognitive maps. J. Exp. Psychol. Hum. Percept. Perform..

[32] Sholl M.J., Acacio J.C., Makar R.O., Leon C. (2000). The relation of sex and sense of direction to spatial orientation in an unfamiliar environment. J. Environ. Psychol..

[33] Hegarty M., Montello D.R., Richardson A.E., Ishikawa T., Lovelace K. (2006). Spatial abilities at different scales: Individual differences in aptitude-test performance and spatial-layout learning. Intelligence.

[34] Piccardi L., Risetti M., Nori R., Thomas J.B. (2011). Familiarity and Environmental Representations of a City: A Self-Report Study. Spatial Memory: Visuospatial Processes, Cognitive Performance and Developmental Effects.

[35] Claessen M.H.G., van der Ham I.J.M. (2017). Classification of navigation impairment: A systematic review of neuropsychological case studies. Neurosci. Biobehav. Rev..

[36] Claessen M.H.G., Visser-Meily J.M.A., Meilinger T., Postma A., de Rooij N.K., van der Ham I.J.M. (2017). A systematic investigation of navigation impairment in chronic stroke patients: Evidence for three distinct types. Neuropsychologia.

[37] Kato Y., Takeuchi Y. (2003). Individual differences in wayfinding strategies. J. Environ. Psychol..

[38] Prestopnik J.L., Roskos-Ewoldsen B. (2000). The relations among wayfinding strategy use, sense of direction, sex, familiarity, and wayfinding ability. J. Environ. Psychol..

[39] Nori R., Piccardi L. (2015). I believe to be good in orienteering… but is that true?. Cogn. Process..

[40] Piccardi L., Cofini V., Palmiero M., Verde P., Boccia M., Palermo L., Guariglia C., Nori R. (2022). Where am I? Searching for the tangle in the developmental topographical disorientation. Neurol. Int..

[41] Bian L., Leslie S.-J., Cimpian A. (2017). Gender stereotypes about intellectual ability emerge early and influence children’s interests. Science.

[42] Evans L. (1991). Traffic Safety and the Driver.

[43] Guerra E., Bernotat J., Carvacho H., Bohner G. (2021). Ladies first: Gender stereotypes drive anticipatory eye-movements during incremental sentence interpretation. Front. Psychol..

[44] Nori R., Piccardi L. (2012). Il senso dell’orientamento: Quanto conta la familiarità con l’ambiente?. G. Ital. Psicol..

[45] Bonavita A., Teghil A., Pesola M.C., Guariglia C., D’Antonio F., Di Vita A., Boccia M. (2022). Overcoming navigational challenges: A novel approach to the study and assessment of topographical orientation. Behav. Res. Methods.

[46] Boccia M., Guariglia C., Sabatini U., Nemmi F. (2016). Navigating toward a novel environment from a route or survey perspective: Neural correlates and context-dependent connectivity. Brain Struct. Funct..

[47] Leys C., Ley C., Klein O., Bernard P., Licata L. (2013). Detecting outliers: Do not use standard deviation around the mean, use absolute deviation around the median. J. Exp. Soc. Psychol..

[48] Kappal S. (2019). Data Normalization Using Median & Median Absolute Deviation (MMAD) based Z-Score for Robust Predictions vs. Min-Max Normalization. Lond. J. Res. Sci. Nat. Form..

[49] Thériault R., Ben-Shachar M.S., Patil I., Lüdecke D., Wiernik B.M., Makowski D. (2024). Check your outliers ! An introduction to identifying statistical outliers in R with easystats. Behav. Res. Methods.

[50] Rousseeuw P.J., Hubert M. (2011). Robust statistics for outlier detection. Wiley Interdiscip. Rev. Data Min. Knowl. Discov..

[51] Teghil A., Boccia M., Bonavita A., Guariglia C. (2019). Temporal features of spatial knowledge: Representing order and duration of topographical information. Behav. Brain Res..

[52] Siegel A.W., White S.H. (1975). The development of spatial representations of large-scale environments. Adv. Child Dev. Behav..

[53] Ishikawa T., Montello D.R. (2006). Spatial knowledge acquisition from direct experience in the environment: Individual differences in the development of metric knowledge and the integration of separately learned places. Cognit. Psychol..

[54] Montello D.R. (1998). A new framework for understanding the acquisition of spatial knowledge in large-scale environments. Spatial and Temporal Reasoning in Geographic Information Systems.

[55] Chrastil E.R., Warren W.H. (2012). Neural evidence for distinct cognitive map and landmark navigation systems. Psychon. Bull. Rev..

[56] Li J., Zhang R., Liu S., Liang Q., Zheng S., He X., Huang R. (2021). Human spatial navigation: Neural representations of spatial scales and reference frames obtained from an ALE meta-analysis. NeuroImage.

[57] Epstein R.A., Higgins J.S., Jablonski K., Feiler A.M. (2005). Visual scene processing in familiar and unfamiliar environments. J. Neurophysiol..

[58] Epstein R.A., Vass L.K. (2014). Neural systems for landmark-based wayfinding in humans. Philos. Trans. R. Soc. B Biol. Sci..

[59] Aguirre G.K., D’Esposito M. (1999). Topographical disorientation: A synthesis and taxonomy. Brain.

[60] Piccardi L., De Luca M., Di Vita A., Palermo L., Tanzilli A., Dacquino C., Pizzamiglio M.R. (2019). Evidence of taxonomy for Developmental Topographical Disorientation: Developmental Landmark Agnosia Case 1. Appl. Neuropsychol. Child.

[61] Schöberl F., Zwergal A., Brandt T. (2020). Testing Navigation in Real Space: Contributions to Understanding the Physiology and Pathology of Human Navigation Control. Front. Neural Circuits.

[62] Bocchi A., Palmiero M., Boccia M., Di Vita A., Guariglia C., Piccardi L. (2020). Travel planning ability in right brain-damaged patients: Two case reports. Front. Hum. Neurosci..

[63] Bryant K.J. (1991). Geographical/spatial orientation ability within real world and simulated large scale environments. Multivar. Behav. Res..

[64] Campbell S.M., Collaer M.L. (2009). Stereotype threat and gender differences in performance on a novel visuospatial task. Psychol. Women Q..

[65] Newcombe N.S., Uttal D.H. (2006). Whorf versus Socrates, round 10. Trends Cogn. Sci..

[66] Spencer S.J., Steele C.M., Quinn D.M. (1999). Stereotype threat and women’s math performance. J. Exp. Soc. Psychol..

[67] Steele C.M. (1997). A threat in the air: How stereotypes shape intellectual identity and performance. Am. Psychol..

[68] Moè A. (2018). Effects of group gender composition on Mental Rotation Test performance in women. Arch. Sex. Behav..

[69] Guizzo F., Moè A., Cadinu M., Bertolli C. (2019). The role of implicit gender spatial stereotyping in mental rotation performance. Acta Psychol..

[70] Cadinu M., Maass A., Rosabianca A., Kiesner J. (2005). Why do women underperform under stereotype threat? Evidence for the role of negative thinking. Psychol. Sci..

[71] Moè A., Pazzaglia F. (2006). Following the instructions! Effects of gender beliefs in mental rotation. Learn. Individ. Differ..

[72] Schmader T., Johns M. (2003). Converging Evidence That Stereotype Threat Reduces Working Memory Capacity. J. Pers. Soc. Psychol..

[73] Walton G.M., Cohen G.L. (2003). Stereotype lift. J. Exp. Soc. Psychol..

[74] Moè A. (2018). Mental rotation and mathematics: Gender-stereotyped beliefs and relationships in primary school children. Learn. Individ. Differ..

[75] Allison C., Redhead E.S., Chan W. (2017). Interaction of task difficulty and gender stereotype threat with a spatial orientation task in a virtual nested environment. Learn. Motiv..

[76] Rosenthal H.E., Norman L., Smith S.P., McGregor A. (2012). Gender-based navigation stereotype improves men’s search for a hidden goal. Sex Roles.

[77] Miola L., Meneghetti C., Pazzaglia F., van der Ham I. (2023). Gender-related differences in environment learning: Examining task characteristics and spatial beliefs. Learn. Individ. Differ..

[78] Coluccia E., Louse G. (2004). Gender differences in spatial orientation: A review. J. Environ. Psychol..

